# Association between stressful life events and psychotic experiences in adolescence: evidence for gene–environment correlations

**DOI:** 10.1192/bjp.bp.114.159079

**Published:** 2016-06

**Authors:** Sania Shakoor, Helena M. S. Zavos, Claire M. A. Haworth, Phillip McGuire, Alastair G. Cardno, Daniel Freeman, Angelica Ronald

**Affiliations:** **Sania Shakoor**, PhD, Centre for Brain and Cognitive Development, Department of Psychological Sciences, Birkbeck, University of London, London; **Helena M. S. Zavos**, PhD, King's College London, MRC Social, Genetic & Developmental Psychiatry Centre, Institute of Psychiatry, Psychology & Neuroscience, London; **Claire M. A. Haworth**, Department of Psychology, University of Warwick, Coventry;, **Phillip McGuire**, PhD, MD, FRCPsych, King's College London, Department of Psychosis Studies, Institute of Psychiatry, Psychology & Neuroscience, London; **Alastair G. Cardno**, PhD, MMedSc, FRCPsych, MBChB, Academic Unit of Psychiatry and Behavioural Sciences, University of Leeds, Leeds; **Daniel Freeman**, PhD, DClinPsy, CPsychol, FBPsS, Department of Psychiatry, University of Oxford, Oxford; **Angelica Ronald**, PhD, Centre for Brain and Cognitive Development, Department of Psychological Sciences, Birkbeck, University of London, London, UK

## Abstract

**Background**

Stressful life events (SLEs) are associated with psychotic experiences. SLEs might act as an environmental risk factor, but may also share a genetic propensity with psychotic experiences.

**Aims**

To estimate the extent to which genetic and environmental factors influence the relationship between SLEs and psychotic experiences.

**Method**

Self- and parent reports from a community-based twin sample (4830 16-year-old pairs) were analysed using structural equation model fitting.

**Results**

SLEs correlated with positive psychotic experiences (*r* = 0.12–0.14, all *P*<0.001). Modest heritability was shown for psychotic experiences (25–57%) and dependent SLEs (32%). Genetic influences explained the majority of the modest covariation between dependent SLEs and paranoia and cognitive disorganisation (bivariate heritabilities 74–86%). The relationship between SLEs and hallucinations and grandiosity was explained by both genetic and common environmental effects.

**Conclusions**

Further to dependent SLEs being an environmental risk factor, individuals may have an underlying genetic propensity increasing their risk of dependent SLEs and positive psychotic experiences.

Studies investigating the aetiology of adolescent psychotic experiences report modest heritability estimates ranging between 33 and 58%, with the remaining variances attributable to environmental influences.^1–3^ Population-based studies of children and adolescents have found that stress-provoking life experiences such as trauma and victimisation are predictive of psychotic experiences.^4^ It is thus reasonable to hypothesise that the same may be true for other stressful life events (SLEs). SLEs are defined as events that require individuals to readjust or experience a change in life.^5^ Literature on SLEs has made a distinction between dependent life events which are typically reliant on an individual's behaviour (such as breaking up with a boy/girlfriend), and independent life events where an individual usually has no control on the occurrence of the event (such as death of a friend or relative).^6^ The relationship between SLEs and psychotic experiences has been explored within the adult population,^7^ with estimates of a fourfold increased risk of psychotic experiences among adults who experienced two SLEs and a sixfold increased risk of psychotic experiences among adults who reported six or more SLEs.^8^ Less, however, is known about the relationship between SLEs and psychotic experiences in adolescents. In one study, researchers found that young adolescents who had more than three SLEs were more likely to experience psychotic experiences.^9^ In another, researchers found that over a 3-year-period, adolescents with a larger number of SLEs had the highest risk of persistent auditory hallucinations.^10^ These observations support the notion that SLEs in general, as well as trauma and victimisation, also contribute towards their risk of psychotic experiences. SLEs are often considered as an index of ‘environmental risk’, yet their heritability has been estimated on average as 28%,^11^ 31% for ‘dependent’ SLEs and 17% for ‘independent’ SLEs.^11^ Since dependent SLEs are more influenced by an individual's behaviour than independent SLEs, they may share a genetic propensity with other heritable behaviours such as psychotic experiences. It is thus feasible to suggest that SLEs are not solely an environmental risk factor for psychotic experiences, but rather that SLEs and psychotic experiences co-occur because of a shared genetic propensity. This possibility needs investigation because the implications for clinical prevention and intervention strategies differ depending on the degree to which the association is driven by genes and the environment. For example if SLEs co-occur with psychotic experiences because of underlying shared genetic influences,^11^ this would indicate the need for future research prevention and intervention strategies to investigate other heritable correlates of psychotic experiences and SLEs such as underlying personality traits.^12–14^

The heritability of ‘environmental’ factors such as SLEs is indicative of gene–environment correlation, whereby genetic factors may in part influence an individual's exposure to specific environments which in turn results in the environmental factors themselves being partly heritable (a gene–environment correlation (*rGE*)).^15^ Assuming an absence of *rGE* by investigating ‘environmental’ risk factors outside of the context of genetic influences may provide a biased estimation of the magnitude of effect an environmental factor has on traits such as psychotic experiences. The investigation of *rGE* contributes to our understanding of environmental risk factors by showing that experiences are in part a result of genetic infleunces,^16^ thus targeting environmental risk factors alone may not be beneficial. In addition to exploring environmental effects directly, this research area demonstrates that focusing attention on underlying pathways through which genetic propensities influence behaviours and traits will also be fruitful. Although to our knowledge no other studies have investigated the genetic and environmental overlap between SLEs and psychotic experiences among adolescents, there is some evidence to suggest that there is a modest degree of genetic overlap between SLEs and depression.^17^ The considerable comorbidity between psychotic experiences and depression^18^ lends support to the hypothesis that some degree of genetic overlap will also be observed between SLEs and psychotic experiences. This is the first study to utilise data from an adolescent twin sample to investigate the genetic and environmental influences contributing to the associations between SLEs and psychotic experiences, as well as the first to assess SLEs in relation to dimensional scales of self-reported psychotic experiences. Our aims were twofold, first to examine whether dependent and independent SLEs are associated with specific psychotic experiences in adolescence, and second, to estimate the extent to which genetic and environmental factors influence the association between dependent SLEs and psychotic experiences.

## Method

### Participants

The Longitudinal Experiences And Perceptions (LEAP) study^19^ is part of the Twins Early Development Study (TEDS) which comprises a community sample of monozygotic (MZ) and dizygotic (DZ) twins born in England and Wales between 1994 and 1996.^20^ 10 874 families from TEDS were invited to take part in the LEAP study. Parent reports for 5076 (46.7%) families and twin reports for 5059 (46.5%) pairs were obtained. Adolescents who participated in the LEAP project had a mean age of 16.32 years. Individuals were excluded (*n* = 327 families) if they did not provide consent at first contact (when TEDS was started), if they had a severe medical disorder, had experienced severe perinatal complications or if their zygosity was unknown. Exclusions for medical disorders included individuals with cystic fibrosis, cerebral palsy, fragile-X syndrome, autism spectrum disorder and those with chromosomal abnormalities such as Down syndrome. After exclusions, the sample reported on in this study comprised 4830 families (44.84% male, 35.94% MZ twin pairs). Comparing the participating and non-participating samples, 94% *v.* 91% were White respectively, and 16% *v.* 12% had mothers with one or more A-levels (UK advanced educational qualification) as their highest qualification respectively.

### Measures

#### SLEs

We assessed SLEs using 20 items from the Coddington Life Events Record.^5^ Parents and adolescents were asked to report on SLEs that had occurred in the past year, by responding ‘Yes’ (1) or ‘No’ (0) to items such as ‘death of a close friend or relative’. Parent and adolescent reports were combined to capture all occurrences of SLEs. This was done using an either/or approach, as simple combination rules work as well, if not better than, more complicated ones.^21,22^ An SLE was scored as ‘Yes’ (1) if either adolescent or parent had reported it. In line with the literature on SLEs,^6,23,24^ a distinction was made between dependent and independent life events. The dependent SLEs scale was the sum of ten items that assessed life events that occur or are potentially likely to arise as a consequence of one's behaviour (i.e. breaking up with a boy/girlfriend). The independent SLEs scale was the sum of ten items that assessed life events that occur or are likely to arise independent of one's behaviour (i.e. death of a friend or relative).

#### Psychotic experiences

Psychotic experiences were assessed using the Specific Psychotic Experiences Questionnaire (SPEQ).^19^ SPEQ assesses specific psychotic experiences as quantitative traits and includes five self-report subscales: paranoia (15 items), hallucinations (9 items), cognitive disorganisation (11 items), grandiosity (8 items), anhedonia (10 items) and one parent-rated subscale: parent-rated negative symptoms (10 items). SPEQ items were derived for the most part from existing scales that were adapted in order for them to be suitable for adolescents.^19^ The subscales were derived from principal component analysis and show good-to-excellent internal consistency (*r* = 0.77–0.93) and test–retest reliability across a 9-month interval (*r* = 0.65–0.74) in this sample. In terms of validity, expert clinical opinion was obtained on the suitability of each item as a measure of adolescent psychotic experiences to ensure content validity.^19^ Furthermore, levels of agreement between scores on SPEQ and the PLIKS (a known measure of psychosis-like symptoms)^25^ showed that adolescents who reported ‘definitely’ having any psychosis-like symptoms on the PLIKS had significantly more psychotic experiences on all the SPEQ subscales (with the exception of anhedonia) when compared with those who did not report any definite psychosis-like symptoms (all significant at *P*<0.001). Positive and cognitive subscales of psychotic experiences showed significant positive correlations with the PLIKS quantitative score (hallucinations *r* = 0.60, paranoia *r* = 0.48, cognitive disorganisation *r* = 0.41, grandiosity *r* = 0.27, all *P*<0.001).^19,25^ Furthermore, for paranoia, cognitive disorganisation, grandiosity and parent-rated negative symptoms SPEQ subscales, individuals who reported a family history of psychosis, as measured by having a first- or second-degree relative with schizophrenia or bipolar disorder, scored higher than individuals without a family history of psychosis (all *P*<0.05).^3^ Further information on the measure can be found in Ronald *et al*.^19^

### Statistical analyses

All analyses were performed using Stata 12 and Open MX. Open MX uses the method of maximum-likelihood estimation and is widely used for analysing genetically sensitive data.^26^ In line with standard behavioural genetics procedure, the effects of gender and age were regressed out, and analyses were conducted using standardised residuals.^27^ Scales of SLEs and psychotic experiences were transformed using square root transformation techniques to reduce skewness and kurtosis and to ensure that the assumption of having a normal distribution was met for genetic modelling (online Table DS1).

### The twin design

The twin design involves MZ and DZ twin pairs to determine the extent to which variation in a single phenotype or covariation between phenotypes are attributable to genetic and environmental influences. Within-pair similarities for MZ and DZ twin pairs were examined separately to establish the role of genetic and environmental influences based on the notion that: (a) MZ twin pairs share 100% of their segregating DNA code and DZ twin pairs share on average 50%; (b) MZ and DZ twin pairs share environmental factors common to both twins in the same family (‘common environment’); and (c) exposure to environmental factors that are experienced differently or are specific to the individual (‘unique environment’) contribute towards differences within twin pairs.^16^

#### Twin analyses

Structural equation modelling techniques were employed to establish the relative importance of additive genetic (A), common environment (C) and unique environmental influences (E) contributing to a phenotype.^16^ This technique further extends to bivariate analyses, by exploring the covariation between phenotypes. The relative contributions of genetic and environmental factors to the association between SLEs and psychotic experiences are referred to as bivariate heritability (*biva*^2^), bivariate common environment (*bivc*^2^) and bivariate unique environment (*bive*^2^). Estimates of covariance between SLEs and psychotic experiences were also used to calculate genetic correlations (*r*_a_), common environment correlations (*r*_c_) and unique environment correlations (*r*_e_), which indexed the extent to which the same set of genes or environments influence both phenotypes.^28^ The relative fit of different models were compared with a saturated model (which provides a full description of the data) to establish the best fitting model for the data.^29^ Parameter estimates were then calculated with confidence intervals using the maximum-likelihood method. The best fitting models were selected based on the lowest Akaike's information criterion (AIC) values. In instances where the AIC values were similar across models (i.e. ACE dropped *r*_a_ and ACE dropped *r*_c_), resulting in the relative influences being difficult to distinguish, the full ACE model was chosen as being the most parsimonious.

### rGE

Further to distinguishing genetic and environmental influences contributing to phenotypic variances and covariances, the twin design also allows for the investigation of *rGE*. Univariate twin models were used to test whether genetic factors influence an ‘environmental’ measure such as SLEs. A genetic influence on an environmental measure would be indicative of *rGE*. Bivariate twin models were also used. Findings suggested *rGE* if genetic factors mediated the association between environmental measures (for example SLEs) and traits (for example psychotic experiences).^16^

## Results

### Phenotypic analyses

Analyses of variance illustrated significant mean effects of gender on psychotic experiences (Table 1). Females reported higher levels of paranoia, hallucinations and cognitive disorganisation, in contrast to males who reported higher levels of grandiosity, anhedonia and had more parent-rated negative symptoms. Females also reported more dependent SLEs than males. No main effect for gender was present for independent SLEs. A main effect for zygosity was observed for paranoia, hallucinations, cognitive disorganisation and parent-rated negative symptoms, whereby DZ twins reported higher levels in comparison to MZ twins. However, the combined effect of gender and zygosity on the means was small (*R*^2^ = 0.00–0.06).

**Table 1:** Means, standard deviations and analysis of variance by gender and zygosity for psychotic experiences and stressful life events (SLEs)

Psychotic experiences	Mean (s.d.)					Score, range	Cronbach, α	Gender, *P*	Zygosity, *P*	ANOVA Gender × zygosity	*R^2^*	*n*
	Total	Male	Female	Monozygotic twins	Dizygotic twins							
Paranoia	12.17 (10.62)	11.75 (10.42)	12.50 (10.77)	11.79 (10.46)	12.38 (10.70)	0–71	0.93	<0.01	0.01	0.45	0.00	4777

Hallucinations	4.65 (6.00)	4.30 (5.77)	4.94 (6.16)	4.47 (5.91)	4.76 (6.05)	0–45	0.87	<0.01	0.01	0.53	0.01	4785

Cognitive disorganisation	3.96 (2.85)	3.40 (2.72)	4.41 (2.87)	3.86 (2.82)	4.01 (2.86)	0–11	0.73	<0.01	0.01	0.66	0.03	4778

Grandiosity	5.32 (4.42)	5.82 (4.56)	4.91 (4.27)	5.26 (4.35)	5.35 (4.46)	0–24	0.85	<0.01	0.56	0.96	0.01	4781

Anhedonia	17.33 (7.93)	19.50 (7.98)	15.58 (7.44)	17.07 (7.96)	17.48 (7.91)	0–50	0.78	<0.01	0.44	0.85	0.06	4781

Parent-rated negative symptoms	2.81 (3.89)	3.17 (4.10)	2.52 (3.69)	2.64 (3.57)	2.91 (4.06)	0–30	0.85	<0.01	0.03	0.02	0.01	4792

Dependent SLEs	1.68 (1.22)	1.66 (1.29)	1.70 (1.16)	1.63 (1.18)	1.71 (1.25)	0–10	0.41	0.01	0.06	0.16	0.00	4782

Independent SLEs	1.58 (1.40)	1.58 (1.42)	1.57 (1.38)	1.53 (1.35)	1.61 (1.43)	0–10	0.42	0.58	0.06	0.02	0.00	4784

a. Means and standard deviations reported prior to transformation, analyses of variances were performed using one random member of each twin pair. Gender *P*-value associated with the effect of gender on the means; zygosity *P*-value associated with the effect of zygosity on the means; gender × zygosity *P*-value associated with the effects of the interaction between gender and zygosity on the means and *R*^2^ is the proportion of the total variance explained by gender and zygosity; *n* is the number of one randomly selected individual from each twin pair.

Phenotypic correlations between SLEs and psychotic experiences are presented in Table 2. Dependent and independent SLEs in adolescence were modestly associated with increased levels of positive psychotic experiences: paranoia, hallucinations, cognitive disorganisation and grandiosity (*r* = 0.12–0.14, all *P*<0.001). Correlations with negative psychotic experiences were low for dependent SLEs (anhedonia *r* = −0.04, *P*<0.05, parent-rated negative symptoms *r* = 0.04, *P*<0.05) and independent SLEs (anhedonia *r* = −0.03, *P*<0.10).

**Table 2:** Phenotypic correlations^a^

	Stressful life events			
	Dependent stressful life events		Independent stressful life events	
Psychotic experiences	*r* (95% CI)	*n*	*r* (95 CI)	*n*
Paranoia	0.14 (0.11 to 0.17)	4732	0.09 (0.06 to 0.12)	4734

Hallucinations	0.14 (0.11 to 0.16)	4740	0.12 (0.09 to 0.15)	4742

Cognitive disorganisation	0.14 (0.11 to 0.16)	4733	0.10 (0.07 to 0.13)	4735

Grandiosity	0.12 (0.10 to 0.15)	4736	0.06 (0.04 to 0.09)	4738

Anhedonia	−0.04 (−0.06 to −0.01)	4736	−0.03 (−0.06 to 0.00)	4738

Parent-rated negative symptoms	0.04 (0.01 to 0.06)	4773	0.08 (0.05 to 0.11)	4775

a. Correlations were performed using one random member of each twin pair. *r*, Pearson's correlation.

The prevalence of SLE and mean scores on specific psychotic experiences scales for individuals with each type of SLE are reported in online Tables DS2–8. For example, the largest effect sizes for paranoia (Cohen's *d* = 0.48) and anhedonia (Cohen's *d* = 0.34) were observed among adolescents who experienced the SLE ‘becoming involved in drugs’. Those who reported ‘being responsible for a road accident’ had the largest effect size for cognitive disorganisation (Cohen's *d* = 0.60). Adolescents who experienced ‘suspension from school/college’ had the largest effect size for hallucinations (Cohen's *d* = 0.36), grandiosity (Cohen's *d* = 0.28) and parent-rated negative symptoms (Cohen's *d* = 0.50).

We did not perform behaviour genetic twin analyses on the independent SLEs measure because these events were family-wide and experienced by both twins within a twin pair. It was therefore not possible to partition variance into genetic and environmental influences. Behaviour genetic analysis of anhedonia and parent-rated negative symptoms with dependent SLEs were not assessed, as phenotypic correlations were considered to be too small (*r* = −0.04 and *r* = 0.04 respectively) to be decomposed into genetic and environmental influences.

### Behaviour genetic analyses

For both psychotic experiences and SLEs, univariate twin correlations (Table 3) were indicative of genetic influences (A), because MZ correlations were consistently larger than DZ correlations. As the DZ correlations were greater than half of MZ correlations, this suggested some common environmental (C) influence. Furthermore, as MZ correlations were less than unity, this implied a moderate unique environmental effect (E).

**Table 3:** Univariate twin and cross-trait cross-twin correlations^a^

ICC (95% CI)		
	Monozygotic twins	Dizygotic twins
Univariate twin correlations, psychotic experiences		
Paranoia	0.52 (0.49 to 0.56)	0.29 (0.24 to 0.34)
Hallucinations	0.43 (0.39 to 0.47)	0.31 (0.26 to 0.35)
Cognitive disorganisation	0.45 (0.41 to 0.48)	0.23 (0.18 to 0.28)
Grandiosity	0.48 (0.44 to 0.52)	0.28 (0.23 to 0.32)
Dependent SLEs	0.52 (0.48 to 0.55)	0.34 (0.30 to 0.39)

Cross-trait cross-twin correlation, psychotic experiences and dependent SLEs		
Paranoia	0.13 (0.08 to 0.17)	0.08 (0.03 to 0.13)
Hallucinations	0.06 (0.02 to 0.11)	0.08 (0.03 to 0.13)
Cognitive disorganisation	0.07 (0.03 to 0.12)	0.04 (−0.02 to 0.09)
Grandiosity	0.13 (0.06 to 0.15)	0.09 (0.04 to 0.14)

SLEs, stressful life events.

a. Correlations were performed using one random member of each twin pair. Intraclass correlations (ICC) using transformed standardised age and gender regressed scales.

Univariate model fitting analyses confirmed initial observations from the twin correlations by showing that genetic (A: 0.25–0.57) and unique environmental (E: 0.17–0.57) factors contributed the most to variances observed in psychotic experiences and dependent SLEs (online Table DS9). All univariate ACE models did not provide a significantly worse fit compared with the saturated models. C could be dropped from the models for paranoia, cognitive disorganisation and anhedonia, and explained small amounts of the variance (0.11–0.26) for the remaining scales.

Bivariate cross-twin cross-trait correlations (Table 3) provided an insight into the extent to which the covariance between dependent SLEs and psychotic experiences was explained by genetic and environmental influences. Collectively, MZ cross-twin cross-trait correlations were larger than DZ cross-twin cross-trait correlations, which is indicative of a genetic influence on the phenotypic associations between SLEs and psychotic experiences. DZ cross-twin cross-trait correlations were somewhat greater than half of MZ cross-twin cross-trait correlations thus implying a modest common environmental effect. Where MZ cross-twin cross-trait correlations were less than the phenotypic correlations between SLEs and psychotic experiences, correlations were suggestive of a unique environmental influence on the covariation.

Results from the bivariate correlated factors solution (online Table DS10) showed that for the association between dependent SLEs and paranoia and cognitive disorganisation scales, the ACE correlated factors solution with dropped *r*_c_ fitted the data best based on the AIC fit index. Analyses (Table 4) demonstrated that the relationship between dependent SLEs and paranoia was almost completely explained by genetic influences (*biva*^2^ = 0.86), with the remaining covariance explained by unique environment. Genetic correlation indicated that a moderate degree of genetic influences overlapped between the two phenotypes (*r*_a_ = 0.33). Furthermore, a small proportion of unique environmental overlap between dependent SLEs and paranoia was also found (*r*_e_ = 0.04). Analyses investigating the association between dependent SLEs and cognitive disorganisation showed a similar pattern whereby high bivariate heritability was found (*biva*^2^ = 0.74). The remaining covariance was explained by unique environment (*bive*^2^ = 0.26). Genetic and unique environment correlations showed that there was modest genetic (*r*_a_ = 0.21) and unique environmental (*r*_e_ = 0.05) overlap between dependent SLEs and cognitive disorganisation.

**Table 4:** Parameter estimates for best fitting bivariate models: proportion of variance explained by genetic and environmental factors^a^

	Dependent stressful life events					
	*biva*^2^ (95% CI)	*bivc*^2^ (95% CI)	*bive*^2^ (95% CI)	*r_a_* (95% CI)	*r_c_* (95% CI)	*r_e_* (95% CI)
Paranoia	0.86 (0.72 to 1.00)	–	0.14 (−0.01 to 0.30)	0.33 (0.24 to 0.45)	–	0.04 (0.01 to 0.09)

Hallucinations	0.44 (−0.22 to 1.00)	0.39 (−0.18 to 0.96)	0.17 (−0.04 to 0.37)	0.18 (−0.09 to 0.46)	0.25 (−0.12 to 0.67)	0.04 (−0.01 to 0.08)

Cognitive disorganisation	0.74 (0.52 to 0.94)	–	0.26 (0.06 to 0.48)	0.21 (0.13 to 0.31)	–	0.05 (0.01 to 0.10)

Grandiosity	0.42 (−0.09 to 0.94)	0.35 (−0.09 to 0.79)	0.23 (0.08 to 0.38)	0.19 (−0.04 to 0.42)	0.38 (−0.12 to 1.00)	0.07 (0.02 to 0.11)

a. Bivariate genetic (*biva*^2^), common environment (*bivc*^2^) and unique environment (*bive*^2^) estimates indicate the proportion of phenotypic correlations explained by genetics, common and unique environment respectively. Bivariate genetic (*r_a_*), common environment (*r_c_*) and unique environment (*r_e_*) correlations indicate the genetic and environmental overlap between psychotic symptoms and stressful life events.

Bivariate analyses further showed that for the association between dependent SLEs and hallucinations and SLEs and grandiosity, the ACE correlated factors solution fitted the data best (online Table DS10). Both genetic and common environmental influences appeared to explain part of the covariance between dependent SLEs and hallucinations, and SLEs and grandiosity (as indicated by the *biva*^2^ and *bivc*^2^ values in Table 4), and both genetic and common environmental influences had some overlapping influences across SLEs and these psychotic experiences (as indicated by the *r*_a_ and *r*_c_ values) but notably the confidence intervals all overlapped with zero. This meant it was not possible to differentiate the relative role of genetic and common environmental influences on the covariance, suggesting they may both play a role. The association between dependent SLEs and grandiosity was also influenced by a modest degree of unique environmental effects (*bive*^2^ = 0.23, Table 4).

## Discussion

Using a community sample of 16-year-old twins, this study showed that SLEs were correlated with positive psychotic experiences (paranoia, hallucinations, cognitive disorganisation, grandiosity) and weakly correlated with negative psychotic experiences. Shared genetic influences explained a substantial proportion of the covariation between paranoia, cognitive disorganisation and dependent SLEs. For hallucinations, and grandiosity, both genes and environment explained some of the covariation with SLEs.

### Are stressful life events associated with psychotic experiences in adolescence?

In our sample of adolescents, females reported more positive psychotic experiences (with the exception of grandiosity) and males reported more grandiosity, anhedonia and had more parent-rated negative symptoms. These findings are similar to those from other cohort-based studies,^30^ and suggest that there may be continuity in gender differences in psychotic experiences among the general population and those with schizophrenia, where males report severer negative symptoms than females.^31^ In keeping with previous studies,^9,10^ having an increased number of dependent and independent SLEs was associated with higher levels of psychotic experiences. This association was stronger for positive (paranoia, hallucinations, cognitive disorganisation, grandiosity) than negative psychotic experiences. Among SLEs, ‘becoming involved in drugs’, ‘suspension from school/college’ and ‘being responsible for a road accident’ were associated with the highest levels of positive psychotic experiences. This specificity of life events is of interest as it is consistent with the association between substance use and psychotic experiences among adolescents.^32^ It also highlights that other correlates such as ‘suspension from school’ may also be of relevance for understanding positive psychotic experiences in adolescence. Collectively, the modest associations reported in this study show that not all adolescents who experience SLEs have psychotic experiences, and vice versa. Experiencing a number of SLEs or specific SLEs such as ‘becoming involved in drugs’ may therefore be a trigger for having elevated levels of positive psychotic experiences.

The association between SLEs and psychotic experiences is consistent with cognitive psychological theories of the development of psychotic experiences,^33^ which suggests that exposure to ‘triggering events’ are particularly damaging in individuals predisposed to disruptions in their cognitive processes. This disruption in cognitive processes in turn may contribute to the risk for psychotic experiences. For example, experiencing an increased number of SLEs may lead individuals to develop cognitive biases that result in viewing their environment to be hostile and threatening. This feeling that ‘the world is out to get me’ may trigger psychotic experiences such as paranoia. Our results inform these models by showing that part of the explanation for individuals having SLEs that co-occur with psychotic experiences is an underlying genetic propensity for both SLEs and psychotic experiences. As we could not examine the temporal relationship between SLEs and psychotic experiences in the present study, it is also possible that adolescents with psychotic experiences may be more likely to have SLEs. For example, experiencing paranoia may lead to being suspicious of others and result in SLEs such as breaking up with a boyfriend or girlfriend. However, evidence from a number of studies has shown life stress (i.e. SLEs) to be a risk factor for psychotic experiences and psychosis among adults and adolescents,^8,10,34,35^ thus supporting the role of SLEs as a catalyst for psychotic experiences such as paranoia.

### To what extent do genetic and environmental factors influence the associations between SLEs and psychotic experiences?

In line with previous research among adolescents within the general population, dependent SLEs and psychotic experiences were in part heritable,^1,2,11,36^ with the remaining variance largely attributable to unique environmental factors. Our findings extend those of previous studies by showing that the relationship between dependent SLEs and psychotic experiences (paranoia and cognitive disorganisation) was almost completely explained by genetic influences. Our findings also provide support for the concept of gene–environment correlation. This can be in one of three ways: active, evocative or passive.^11^ Gene–environment correlations could be ‘active’, whereby the genetic propensity that leads individuals to seek out situations resulting in dependent SLEs is the same genetic influence that increases the risk for psychotic experiences (paranoia and cognitive disorganisation). Alternatively, it could be ‘evocative’, whereby dependent SLEs, which are partly genetically influenced, result in environments or incite behaviours from others that result in elevated levels of paranoia and cognitive disorganisation. Finally, gene–environment correlations may be ‘passive’, whereby genetic factors that increase the likelihood of dependent SLEs on the part of the parent are shared with adolescents through the environments parents raise them in, and in turn are associated with psychotic experiences. Focusing on ‘environmental’ factors in isolation may not therefore be an optimal research strategy. Examining factors through which a genetic vulnerability for having dependent SLEs and psychotic experiences are translating into behaviours (such as home environment or parenting), may help in identifying underlying mechanisms contributing towards psychotic experiences and SLEs among adolescents. For the relationship between dependent SLEs and hallucinations, and grandiosity, both genetic and common environmental influences appeared to play a role but their relative role was not clear.

### Limitations and strengths

The study's cross-sectional design did not make it possible to test for temporal priority. Therefore, although interpretations were in the direction of SLEs leading to psychotic experiences, it is possible that psychotic experiences may have altered individuals' behaviours resulting in the SLEs being reported here. Furthermore, as participants were asked to report on their psychotic experiences from the past month and SLEs from the past 12 months, there may be recall bias, whereby SLEs were more difficult to remember given that the reporting period was more distal. Second, we used self-reports of paranoia, hallucinations, cognitive disorganisation, grandiosity and anhedonia. This work could be replicated using in-depth interviews and reports from other informants. Third, we observed modest correlations between SLEs and psychotic experiences. Estimates of bivariate heritability and environmental influences reported in this study are therefore explaining a small proportion of variance within psychotic experiences.

This study also has a number of strengths. It is the first to investigate psychotic experiences and SLE among a large community sample of adolescents, an age just prior to the modal age at onset of psychotic disorders such as schizophrenia. Furthermore, the genetically informative study design allowed the relationships to be decomposed into genetic and environmental influences. In contrast to other studies that have focused on a specific type of psychotic experience (i.e. hallucinations^37^), this study included multiple informant reports of specific psychotic experiences, which were measured as dimensions and included both positive and negative psychotic experiences. Adolescents reported on paranoia, hallucinations, cognitive disorganisation, grandiosity and anhedonia and parents reported on negative symptoms.

### Implications

Our work underlines the importance of viewing certain environmental risk factors within the context of genetics. It highlights the importance of not always categorising risk factors as either environmental or genetic as they may be a combination of the two. Our finding of a shared genetic propensity between SLEs and paranoia and cognitive disorganisation could help research and interventions focus on other types of (heritable) behaviours shown developmentally earlier (i.e. impulsivity), which may jointly increase the risk of psychotic experiences and dependent SLEs. Moreover, as DNA does not change throughout the life course, a shared genetic propensity between SLEs and psychotic experiences would imply that clinical intervention should take into account the continued vulnerability of individuals with psychotic experiences to have dependent SLEs. Further research is needed, but the results are suggestive that focusing on and dampening the effects of common environmental risks that contribute towards SLEs might decrease the risk of psychotic experiences such as hallucinations in vulnerable individuals.

SLEs are associated with positive psychotic experiences in adolescence. This is via a shared genetic propensity in addition to the more recognised mechanism of shared environment risk. An accurate understanding of the mechanisms by which risk factors increase the risk for psychotic experiences is imperative for improving intervention and prevention strategies in adolescence.
